# Invasive cancers are not necessarily from preformed *in situ* tumours — an alternative way of carcinogenesis from misplaced stem cells

**DOI:** 10.1111/jcmm.12078

**Published:** 2013-06-07

**Authors:** Rui-An Wang, Zeng-Shan Li, Hui-Zhong Zhang, Ping-Ju Zheng, Qin-Long Li, Jian-Guo Shi, Qing-Guo Yan, Jing Ye, Jing-Bo Wang, Ying Guo, Xiao-Feng Huang, Ying-Hao Yu

**Affiliations:** a State Key Lab of Cancer Biology, The Fourth Military Medical UniversityXi'an, Shaanxi Pr., China; b Department of Pathology of Xijing Hospital, The Fourth Military Medical UniversityXi'an, Shaanxi Pr., China; c Department of Pathology and Pathophysiology, The Fourth Military Medical UniversityXi'an, Shaanxi Pr., China; d Department of Laboratory Medicine of Tangdu Hospital, The Fourth Military Medical UniversityXi'an, Shaanxi Pr., China; e Department of Oncology, Chang'an HospitalXi'an, Shaanxi Pr., China; f Department of Pathology, Fuzhou General Hospital of Nanjing Commander Military RegionFuzhou, Fujian Pr., China

**Keywords:** Carcinogenesis, stem cell misplacement theory, basement membrane, breast cancer, DCIS, stroma, *quasi*-cancer

## Abstract

Cancers are thought to be the result of accumulated gene mutations in cells. Carcinomas, which are cancers arising from epithelial tissues usually go through several stages of development: atypical hyperplasia, carcinoma *in situ* and then invasive carcinoma, which might further metastasize. However, we think that the present pathological data are enough to prove that there might be an alternative way of carcinogenesis. We propose that majority of invasive cancers arise in the connective tissue stroma *de novo,* from the misplaced epithelial stem cells which come to the wrong land of connective tissue stroma by accident. The *in situ* carcinomas, which are mostly curable, should not be considered genuine cancer, but rather as *quasi-cancer*. We design this new theory of carcinogenesis as the stem cell misplacement theory (SCMT). Our SCMT theory chains together other carcinogenesis theories such as the inflammation-cancer chain, the stem cell theory and the tissue organization field theory. However, we deny the pathway of somatic mutation theory as the major pathway of carcinogenesis.

## The invasive cancers do not all come from the *in situ* carcinomas

**Fig. 1:**
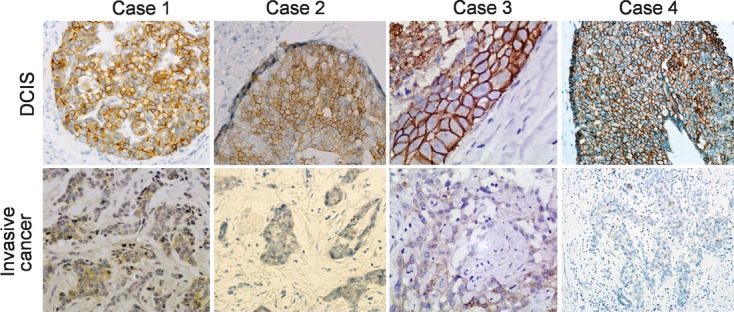
HER2 overexpression in four cases of coexisting ductal carcinoma *in situ* (DCIS) and invasive breast carcinoma. DCIS shows strong staining at a score of 3, while the invasive cancer parts are basically negative.

In breast tumour pathology, there is a well-known perplexing phenomena in which the ductal carcinoma *in situ* (DCIS) often shows a much higher frequency of HER2 gene amplification and overexpression than that of the invasive breast cancer (50–60% *versus* 20–30%) 1–7. And it is often seen that in the same cases of invasive breast cancer mixed with DCIS, the DCIS portion shows HER2-positive immunostaining, while the invasive cancer cells are HER2-negative (Fig. 1). It is paradoxical in a way that HER2 is a powerful oncogene whose overexpression promotes the progression of the tumour and is linked with a poorer clinical outcome in the invasive breast cancer 8, 9, but here the DCIS with HER2 overexpression seems to have no advantage over those of HER2-negative to further develop into invasive breast cancer.

Then is it possible that the invasive cancers have origins other than the *in situ* carcinoma? We looked for other supporting evidence. We found that in the statistical data of breast epidemiology that, although the incidence of the DCIS has increased about 16 times over the last 30 years (32/100,000 individuals in United States) 10, it is still much lower than that of the invasive breast cancer (around 130/100,000 individuals) 10. If we take those previous studies which showed that the diagnosed DCIS without any treatment takes 5–30 years or more to develop into invasive cancer as true 11, then the incidence of DCIS should be much higher than that of the invasive breast cancers, which is the reverse case here. We went over through the archived slides of the invasive breast cancers of the last 2 years. Of about 500 cases total, we found DCIS components in less than half, which is within the same range of those previously reported 12.

Another type of breast tumour also came to our attention: the lobular carcinoma *in situ* (LCIS). When LCIS is diagnosed, usually there is no treatment needed other than close monitoring follow up. That is because if a LCIS is found, the bilateral breasts both have equally increased risk of developing invasive breast cancer at a rate of 27% 13. And in those cases that developed malignant tumours later on, the number of invasive ductal carcinoma is much more than that of the invasive lobular carcinoma. This suggests that, in the case of LCIS, the patient has increased cancer risk, but LCIS does not develop towards invasive cancer *per se* 13. By this, where then does the invasive lobular carcinoma come from?

Putting these lines of evidence together, we think it is proper that we should rethink the current carcinogenesis theory of gene mutation. This theory holds that epithelial cells accumulate gene mutations first, and then transformation occurs, followed by abnormal proliferation of the transformed cells in the epithelium, and further develop into a stage of what is called *in situ* carcinoma 14. Here, we propose a new theory of carcinogenesis, in which we think that a considerable portion of the invasive cancers arise directly in the connective tissue stroma *de novo,* without preformed cancerous lesions in the epithelium. The seed of the invasive cancer is a misplaced stem cell which lands in the wrong location (stroma) in error from the damaged basement membrane. We refer to the new theory as SCMT, in contrast to the current dominant somatic cell gene mutation theory (SMT).

## The true cancer develops *de novo* from a misplaced epithelial stem cell

We believe at least some invasive cancers formed *de novo* in the stromal tissue without necessarily having precursor lesions of *in situ* carcinoma or dysplasia. The seed of the cancer is a misplaced epithelial stem cell which lands to the wrong location of stromal connective tissue by accident. When a stem cell undergoes mitosis, it rounds up, loses connections with its neighbouring cells and becomes coated with a film of hyaluronan (HA) 15. The HA confers cells with the ability of moving freely due to its structure. And it is at this time the basement membrane (BM) beneath the stem cell undergoing mitosis experiences some break ups. As the saying goes, ‘cancer cells are nothing special, they just did usual things at unusual times’ 15. The seed of the cancer is a misplaced epithelial stem cell which lands in the wrong location. The stem cell then comes to this new territory by mistake through the leakage of the BM. In support of this notion, we found in the literature that an inheritable defect of Type VII collagen which is an important component of skin basement membrane is associated with increased incidence of invasive skin cancer 16.

**Fig. 2:**
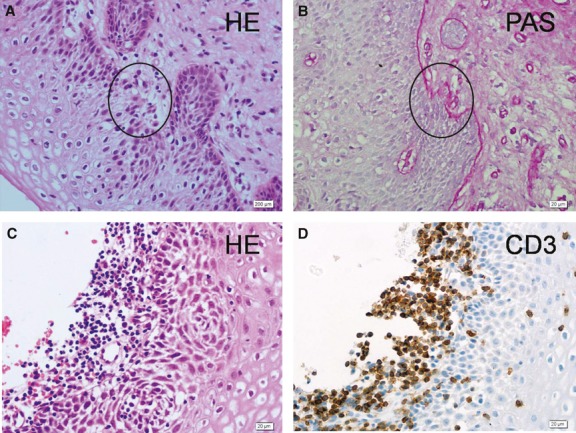
Inflammation destroys basement membrane. Sections of uteral cervix. (**A** and **B**) These are adjacent sections. In the circled region, there is inflammation (**A**), which may have damaged the basement membrane, as shown in (**B**) by PAS staining. (**C** and **D**) These are adjacent sections, which show intense inflammation with possible damage of basement membrane.

Because it is not their own territory, naturally these wrongly placed stem cells would be seen as alien invaders by the immune system. But time and again it happens. And one day, one of these stem cells in the wrong land managed to escape the immune system and survived. Inflammation, trauma damaged BM (Fig. 2) and/or ageing, degeneration of BM, all these situations increase the chances of the stem cell invasion, and these accumulated invasions add up to the increased probability of developing cancer.

There are pros and cons being in the stroma for these mislanded stem cells. The bad thing is that the immune system will be constantly going after them. The good thing is, the stroma is rich in hyaluronan, the ligand for CD44, which the stem cells constantly bear on their cell membranes 15. By the law of the Darwinian quest for survival, these misplaced stem cells must first strive to divide to increase their population. However, they cannot normally differentiate in this wrong place as they do in the epithelium above the BM, because of the inappropriate milieu. Consequently, there is much more increased cell proliferation as shown by mitotic figures and Ki-67 labelling indexes. As we recently proposed, increased apoptosis drives the cells proliferate 17. At the very beginning, the mislanded stem cell is neither a genetically mutated nor a biologically transformed *malignant* cell. However, in the process of combating the tough environment for survival, the colony of the cells from this stem cell accumulates an increasing number of mutations. The possible underlying mechanism is, to quickly expand their population, that they nullify the tumour suppressor genes such as p53. If a person has a previous existing null mutation in one of the alleles, it would be much easier to do so. This process is called loss of heterozygosity. The loss of functional mutation of p53, which checks for the accuracy of gene duplication, leads to increasing amounts of mutations, and the genomic instability of tumour. The mutation is random. This explains why after so many years of whole genomic sequencing of a variety of tumours, no fixed set of common mutations has been found across the tumour spectrum, or even in the same type of tumour 18. Thus, this novel SCMT carcinogenesis theory of stem cell misplacement explains better the gene mutation than SMT, which holds that gene mutation accumulation leads to the transformation of the epithelial cells. There is no explanation of how all these mutations happen and what mutation is needed for the transformation to happen in the SMT theory.

**Fig. 3:**
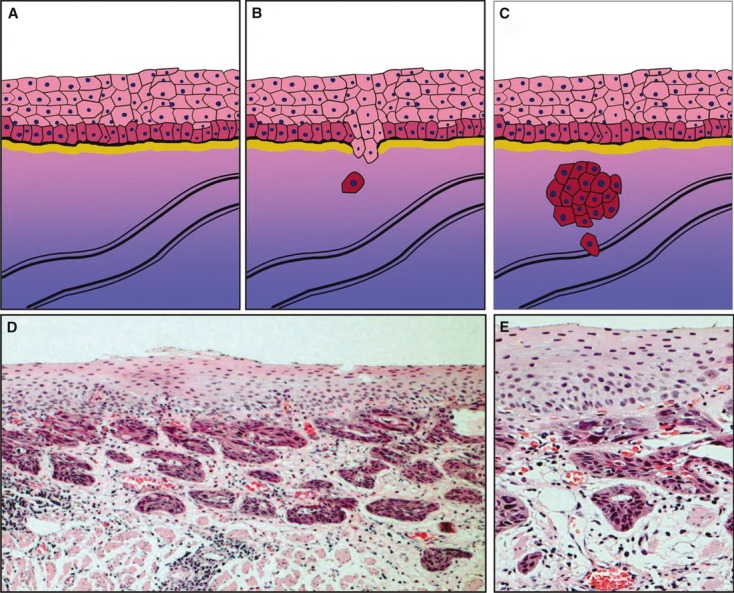
Schematic diagram shows the process of carcinogenesis beginning from the mislanding of an epithelial stem cell, and an actual case of early oesophageal cancer. (**A**) Normal epithelium. (**B**) Basement membrane damage results in the misplacement of a stem cell in the stroma. (**C**) The misplaced stem cell develops into invasive cancer. (**D**–**E**) a case of early oesophageal cancer. Invasive cancer tissues can be seen under the almost normal squamous epithelium. Clinically, the patient was precluded from cancer metastasis.

The novel carcinogenesis process by SCMT is depicted in Figure 3, supported with an actual case of early oesophageal cancer which shows almost normal epithelium above the cancer tissue.

## HA overcoat on the stem cells confers them the ability of metastasis

The epithelial–mesenchymal transition (EMT) theory of cancer metastasis is facing increasing doubts, especially from pathologists 19, 20. In our new theory, the misplaced stem cells are capable of metastasis as it is, with no need of any further modification or extra specific set of gene expression. This innate ability 21 of the stem cells may be related to the CD44 which is now a known marker of the stem cells. CD44 is the known receptor for HA, which is quite rich in stroma, especially in the peri-tumour stroma 15. The film of HA coat on the stem cells confers upon them the ability to migrate easily in a stealthy way 15. Thus, as the way it is put, cancer is the price paid for metazoan evolution 15. Before the stage of metazoan, no HA but chondroitin is produced and cells are not movable within the body 15. This also explains the clinical phenomena of the super early metastasis of some cancers, even at a stage before the primary cancer was detected. In fact, the amount of cancer stromal HA as well as the cancer cell-secreted HA have been linked with poor prognosis of patients 22, 23.

## Telomerase may have never been lost in the stem cells, so it does not need to be reactivated in cancer

One of the important features of cancer cells is their ability of limitless proliferation 24. The mechanism underlining this biological phenomenon is that they can overcome the telomere shortening by telomerase. For a long period of time, scientists wonder and try to elucidate the mechanism of telomerase reactivation. In the SCMT theory, we think the tumours may have never lost their telomerase activity rather than it was lost and regained. Most somatic cells lost their telomerase soon after birth. However, telomerase is retained in a considerable portion of stem cells to maintain their proliferative activity 25. Therefore, starting from stem cells, the carcinogenesis process would be much simpler than from those differentiated cells.

## Testing SCMT

We have to acknowledge that a big science theory is not easy to prove. So far, the SMT theory which dominated our cancer research has not been vigorously tested although most individuals believe it. Herein, we designed an experiment which could be used to test the validity of our SCMT theory.

**Fig. 4:**
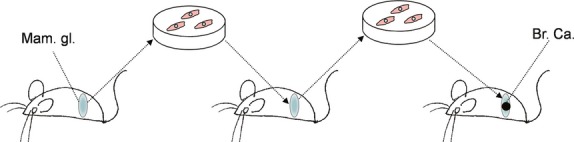
Testing of the stem cell misplacement theory (SCMT) with serial transplantation experiment. Donor mouse mammary epithelial cells with special marker are to be isolated and transplanted into the mammary glands of recipient mice. After several weeks, the mammary gland epithelial cells of recipient mice will be again isolated, labelled cells sorted and subsequently transplanted to next animal. Tumours are expected to show up after several rounds of transplantation.

An experiment which would directly show that the misplaced epithelial stem cells develop into cancer is to inject the isolated stem cells into the stroma and to see if cancer would occur. However, as the common laboratory animals like mice and rats usually just live 2.5 years and most tumours seen in these animals are lymphomas, which are different from that of human beings, the chance of success with this experiment would be very low. To overcome this shortcoming, a serial transplantation strategy can be adopted (Fig. 4).

To start the experiment, genetically modified mice with specific labelling such as GFP or β-gal will be used as stem cell donor animal and the same inbreed mice without labelling molecular marker will be used as the recipient animal. Stem cell containing epithelial cells from donor mouse mammary glands can be isolated and injected to the mammary gland fat pads of recipient animal. After several weeks, the mammary glands containing transplanted cells are to be taken and epithelial cells are to be isolated and sorted by fluorescence, and transplanted to next animal. After several rounds of transplantation, tumours can be expected to arise from some animals.

By this experimental strategy, the TOFT theory will also be tested. That is, if the donor and recipient animals are grouped by younger and old animals, we expect that cancers would show up earlier in the old recipient animal group.

In summary, we have come up with a new theory of carcinogenesis: we believe that the majority of invasive cancers arise in the connective tissue stroma *de novo* rather than from preformed cancerous epithelial lesions. In mammary glands, this way of carcinogenesis could account for more than half of invasive cancers. In this new theory of carcinogenesis, the damage of basement membrane is the key event by which stem cells come to the wrong land by mistake (Fig. 3). There is no transformation or gene mutation required of the stem cells before they come to the stroma. The gene mutation is acquired and accumulated later, during the process of the tumour growth in the stroma. Inflammation and ageing increases cancer incidence by damaging the BM or may be linked with the wearing out of the BM. There is no need of EMT for metastasis to happen, and telomerase is not reactivated as it has never been lost. *In situ* carcinomas, which are increasingly seen more these days, are not mandatory stages of invasive cancer development. As *in situ* carcinomas are mostly curable, we think that they should not be regarded as genuine cancer, but rather, *quasi-cancer*. This is very important as the word ‘cancer’ easily overwhelms patients and doctors are used to over treating their patients with *in situ* carcinomas. This theory is logically simple and provides good connections between other theories of carcinogenesis such as inflammation-cancer chain, cancer stem cell theory and tissue organization theory 26. This new theory may open a new horizon for elucidation of the mechanism of cancer, both development and progression, *versus* prevention and treatment.
